# Changes in allelic imbalances in locally advanced breast cancers after chemotherapy

**DOI:** 10.1038/sj.bjc.6603937

**Published:** 2007-09-18

**Authors:** M Varna, H Soliman, J-P Feugeas, E Turpin, D Chapelin, L Legrès, L-F Plassa, A de Roquancourt, M Espié, J-L Misset, A Janin, H de Thé, P Bertheau

**Affiliations:** 1 INSERM U728, University Hematology Institute, University Paris 7 Denis Diderot, Paris, France; 2 Department of Biochemistry, Hospital Saint-Louis APHP, 1 av. C. Vellefaux, 75010 Paris, France; 3 CNRS UMR 7151, University Hematology Institute, University Paris 7 Denis Diderot, Paris, France; 4 Department of Pathology, Hospital Saint-Louis APHP, 1 av. C. Vellefaux, 75010 Paris, France; 5 Department of Oncology, Hospital Saint-Louis APHP, 1 av. C. Vellefaux, 75010 Paris, France

**Keywords:** loss of heterozygosity, breast cancer, allelic imbalance, TP53, chemotherapy

## Abstract

In advanced breast cancers, *TP53* mutation is highly predictive of complete response to high-dose epirubicin/cyclophosphamide chemotherapy. In these tumours with an altered control of genomic stability, accumulation of chemotherapy-induced genetic alterations may contribute to cell death and account for complete response. To explore the effects of chemotherapy on stability of the tumour genome, allelic profiles were obtained from microdissected tumour samples before and after chemotherapy in 29 unresponsive breast cancers (9 with *TP53* mutation). Ninety-four per cent allelic profiles remained unchanged after treatment. Interestingly, 11 profiles (6%) showed important changes after treatment; allelic imbalances significantly increased (four cases) or decreased (seven cases) after chemotherapy in three distinct experiments, two of which using laser microdissected tumour cells. These genetic changes were not linked to the *TP53* status, but one tumour showed complete disappearance of *TP53*-mutated cells in the residual tumour after treatment. Altogether, these observations carry important implications for the clonal evolution of breast cancers treated with DNA-damaging agents, as they point both to the importance of tumour heterogeneity and chemotherapy-driven selection of subclones.

Breast cancers are a heterogeneous group of tumours. Whereas most advanced breast cancer patients receive neoadjuvant chemotherapy, less than 20% of them will fully benefit from this type of treatment and reach complete pathological response, which is closely linked with longer survival (Bertheau *et al*, 2005; Kaufmann *et al*, 2005). The identification of predictive markers of response to chemotherapy is therefore a critical challenge for advanced breast cancer patients.

In previous studies (Bertheau *et al*, 2002, 2007), we have shown in non-inflammatory locally advanced breast cancers that *TP53* mutations are highly predictive of complete pathological response to high-dose epirubicin/cyclophosphamide chemotherapy. TP53 is known to control genomic integrity (Vogelstein *et al*, 2000), and is induced by cytotoxic drugs (Lowe *et al*, 1993; Bunz *et al*, 1999), whereas its potential role as a prognostic and a predictive marker in cancer patients remains controversial (Vousden and Prives, 2005; Berns, 2006). We hypothesised that breast tumours with *TP53* mutations could accumulate genetic alterations leading to complete tumour response through mitotic catastrophes and tumour cell death (Huang *et al*, 2005). Whereas obviously, we cannot explore the genome of tumours that underwent complete response, those that were not eradicated can be analysed. Note that we have previously shown that *TP53* mutant tumours that did not undergo complete remissions have a worse prognosis than the other groups (Bertheau *et al*, 2007). This might be caused by chemotherapy-induced additional genetic changes that favour progression.

To test this hypothesis, we further explored the effects of chemotherapy on the tumour genome. Early tumour genetic changes after chemotherapy have been poorly studied in patients. We analysed here, in 29 locally advanced breast cancers, the allelic profiles obtained with ten polymorphic microsatellite markers known to be often involved in breast carcinoma (Bertheau *et al*, 2001). Allelic profiles obtained after chemotherapy were compared to those obtained before, using laser-microdissected surgical specimen.

## PATIENTS AND METHODS

### Patients and tumours

Among 80 non-inflammatory locally advanced breast cancers treated in a single institution from 1997 to 2001 with first-line chemotherapy and already studied for their *TP53* status and response to treatment (Bertheau *et al*, 2007), we included 29 patients who fulfilled all the following criteria: patient informed of the study according to our Institutional Review Board recommendations; identical treatment (6 cycles of a dose–dense regimen associating 75 mg m^−2^ epirubicin and 1200 mg m^−2^ cyclophosphamide, given every 14 days with G-CSF support in case of febrile neutropenia; Cottu *et al*, 1999); and incomplete pathological response to treatment. The patients were women aged 23 to 87 (median age 43). Patients and tumours characteristics are given in Table 1. TNM tumour stages are given according to the WHO criteria (Sobin, 2002) and tumour grades are given according to the modified Scarff Bloom and Richardson system (Elston and Ellis, 1991). ESR1 and ERBB2 status were determined with immunohistochemistry (primary antibodies, respectively, clone 6F11 and clone CB11, Novocastra, Newcastle, UK). All patients underwent an initial open incisional biopsy, followed by chemotherapy and, three months after diagnosis, mastectomy and axillary lymph node dissection were performed. Only tumours with at least 5000 remaining cells on the mastectomy specimen tissue sections were included in the analysis. Three different categories of incomplete pathological response were individualised according to a methodology previously validated (Bertheau *et al*, 2005): tumours with post-treatment cellular density less than 50% compared to pre-treatment tumours defined ‘major pathological response’, tumours with post-treatment cellular density only slightly decreased, but with post-treatment cellular alterations defined ‘minor pathological response’, whereas tumours that remained unchanged after treatment defined ‘absence of response’.

### TP53 typing

After tumour RNA extraction (Chomczynski and Sacchi, 1987), *TP53* status was determined by the yeast functional assay (Flaman *et al*, 1995; Waridel *et al*, 1997). Tumours were considered *TP53* mutant when (i) more than 15% of the yeast colonies were red and (ii) analysis using the split versions of the test could identify the defect in the 5′ or 3′ part of the gene, confirming the initial determination and (iii) sequence analysis from mutant yeast colonies could identify an unambiguous genetic defect (mutation, deletion, splicing defects…).

We determined the *TP53* status with the yeast functional assay in all 29 tumours before and after chemotherapy, whenever sufficient frozen material was available for RNA extraction (18 tumours).

For the nine cases with *TP53* mutation before treatment, frozen tumour tissue was available after treatment in six cases and used to perform the yeast functional test as well as *TP53* gene sequencing. For the other three mutated cases, we tried to use formalin-fixed paraffin-embedded tissue, but failed to obtain good enough RNA to perform the yeast functional assay or long enough DNA fragments (>300 bp) to perform gene sequencing.

For all nine mutated cases, TP53 immunostainings on paraffin-embedded samples were obtained before and after treatment using DO-7 monoclonal mouse anti-human antibody (Dako, Trappes, France), diluted 1/50 in an automated immunostainer (Ventana Medical Systems SA, Illkirch, France). The percentage of stained cells (nuclear staining) was noted.

### Laser-microdissection and pressure catapulting (LMPC)

**Figure 1:**
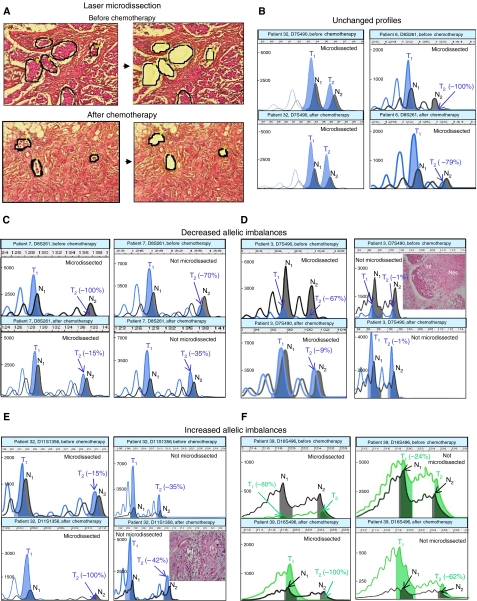
(**A**) Laser-microdissection of breast carcinoma paraffin sections stained with H&E. The left panels show the selected areas (surrounded by a black line) before microdissection and the right panels show the same areas after microdissection. Tumour cell density is much lower after chemotherapy (lower panels). (**B**) Examples of unchanged profiles in two patients: retention of heterozygosity (left panels) or loss of heterozygosity (right panels) before and after chemotherapy. (**C** and **D**) Examples of decreased AI in two patients. (**C**) For patient 7 marker D8S261, LOH is present before chemotherapy with complete allelic loss on microdissected tumour (left panel) and important AI on not microdissected tumour (right panel). After chemotherapy, AI is strongly reduced with both methods (lower panels). (**D**) For patient 3 marker D7S490, LOH is present before chemotherapy on microdissected tumour (left upper panel) and LOH has disappeared after chemotherapy (left lower panel). Use of not microdissected tissue is not conclusive since tumour cells are contaminated with numerous inflammatory cells (inset: tumour cells (T), inflammatory cells (Inf), necrosis (Nec)). (**E** and **F**) Examples of increased AI in two patients. (**E**) For patient 32 marker D11S1356, there is no AI before chemotherapy with both microdissected and not microdissected tumours (upper panels), whereas complete LOH is present after chemotherapy with microdissected tumour (lower left panel). Use of whole tumour tissue after chemotherapy (lower right panel) is not conclusive (does not reach -50%), as tumour cells are contaminated with inflammatory cells (inset: tumour cells (T), inflammatory cells (Inf)). (**F**) For patient 39 marker D16S496, LOH is present on the short allele before chemotherapy and on the long allele after chemotherapy with microdissected tumour (left panels). Profiles with whole tumour tissue (right panels) are close. For all allelic profiles, normal DNA profiles are shown in black (alleles N1 and N2), tumour DNA profiles are shown in colour (blue or green) (alleles T1 and T2). *x*-axis shows allelic size in base pairs, *y*-axis shows fluorescence intensity.

Using a PALM Microbeam/Olympus system, LMPC was performed on all 29 tumours before and after chemotherapy: 7 *μ*m thick paraffin sections were spread on membrane-coated slides and stained with H&E (Figure 1A). A pulsed UV-A nitrogen laser (337 nm) was used to cut and catapult small tissue fragments directly into the buffer-containing cap of a microfuge tube. For each tumour, at least 5000 cell sections obtained in several tumour areas were lysed in 30 *μ*l of buffer (50 mM Tris-HCl (pH 7.5), 1 mM EDTA, 0.5% Tween 20, 0.2 mg ml^−1^ proteinase K). After an incubation of 24–48 h at 56°C, proteinase K was inactivated at 95°C for 10 min. No further DNA extraction was performed before PCR analysis for these microdissected samples (Bertheau *et al*, 2001).

### DNA extraction

Control allelic profiles were obtained with DNA extracted from blood cells: after cell lysis and proteinase K degradation, DNA was obtained with phenol chloroform extraction followed by DNA precipitation with ethanol and solved in TE buffer (Tris 10 mM (pH 7.5), EDTA 1 mM) (Muniz *et al*, 1994).

Tumours that showed post-treatment allelic changes with microdissected samples (see Results) were also analysed with DNA extracted from frozen whole tumour tissue. Briefly, frozen tumour sections were immersed in a buffer containing 8 M urea, 0.3 M NaCl, 10 mM EDTA, 2% SDS and 10 mM Tris-HCL, pH 7.5, and then submitted to phenol chloroform DNA extraction.

### PCR

For microdissected cells, a volume of lysis buffer accounting for 500 cells was added in each PCR vial. For DNA extracted from blood cells, 10 ng DNA was used for each PCR. The following 10 microsatellite dinucleotide repeats were used (Table 2): D3S1573; D7S490; D8S1820; D8S261; D11S860; D11S1356; D13S171; D16S496; IGP53; and D17S855. The PCR mix contained 1 U Taq Gold (Applied Biosystems, Foster City, CA, USA), 2.5–4 mM MgCl_2_, 0.2 mM dNTP, 0.2 *μ*M labelled forward primers (NED™ for normal, FAM (6-carboxyfluorescein) or VIC™ for tumour) and 0.2 *μ*M non-labelled reverse primers. The PCR final volume was 20 *μ*l. Thirty-five cycles of PCR were performed.

After denaturation, the PCR products were run on ABI PRISM 310 Genetic Analyzer. The analysis of the migration data were performed with Genescan 3.1 software (Applied Biosystems).

### Allelic profiles analysis

Fluorescent allelic ratios obtained from microdissected tumour tissue were compared with fluorescent allelic ratios obtained from control DNA, allowing the measurement of allelic imbalances (AI). Loss of heterozygosity (LOH) was defined as AI greater than 50% for one tumour allele.

AI observed after chemotherapy were then compared to AI observed before chemotherapy, showing locus with unchanged AI, decreased AI or increased AI. Decreased or increased AI were considered significant only when allelic peak heights differed by at least 50%. This threshold was chosen to minimize the risk of false-positive results induced by PCR variations. All these significant pre-post-treatment differences were verified with a second round of PCR performed with microdissected tissue and a third round of PCR performed with frozen whole tumour DNA.

## RESULTS

### Allelic profiles before chemotherapy

Among a total of 290 ‘before chemotherapy’ PCR (10 markers performed in 29 tumours), 63 allelic profiles were homozygote (non-informative) and 15 PCR could not be analysed due to technical issues. The rates of informativity for these markers were similar to those expected from databases (Tables 2 and 3). The 212 informative profiles revealed 87 (41%) LOH and 125 (59%) retentions of heterozygosity. Rates of LOH at each locus were similar to those observed in the literature in the corresponding chromosomal regions (Kerangueven *et al*, 1997; Braga *et al*, 1999; Katsama *et al*, 2000; Osborne and Hamshere, 2000; Shen *et al*, 2000; Hirano *et al*, 2001; Miyakis and Spandidos, 2002; Wang *et al*, 2004; Mao *et al*, 2005).

### Allelic profiles after chemotherapy

Among a total of 290 ‘after chemotherapy’ PCR (29 tumours, 10 markers), 63 allelic profiles were homozygote (non-informative) and 24 PCR could not be analysed due to technical issues. The 203 informative profiles revealed 78 (38%) LOH and 125 (62%) retentions of heterozygosity. (Tables 2 and 3)

### Comparison of allelic profiles before and after chemotherapy

Altogether, 191 pairs of PCR were informative and analysable before and after chemotherapy. In 180 pairs (94%), allelic profiles before and after chemotherapy were not different (Figure 1B; Table 3, green cells). However, in 11 pairs, pre- and post-chemotherapy profiles were significantly different. In seven pairs (4%), AI observed before chemotherapy had significantly decreased after chemotherapy (Figure 1C and D; Table 3, blue cells). In two pairs (1%), almost normal profiles observed before chemotherapy were clearly imbalanced after chemotherapy (Figure 1E; Table 3, orange cells). In 2 pairs (1%), AI observed for one allele before chemotherapy was found for the other allele after chemotherapy (Figure 1F; Table 3, orange^*^ cells).

For these 11 important changes detected after chemotherapy, we reproduced once the analysis with microdissected samples. A further analysis with non-microdissected extracted DNA showed very close results (Figure 1C and F) in five pairs, and non-conclusive results (Figure 1D and E) in the other six pairs.

### Allelic profiles and TP53 status

The overall rates of LOH at all loci were similar in tumours with or without *TP53* mutations, before (47 and 38%, respectively) or after treatment (41 and 37%, respectively).

LOH at the *TP53* intragenic IGP53 locus before treatment was observed in 4 out of these 9 tumours with *TP53* mutation (44%) and in 5 out of 20 tumours without *TP53* mutation (20%). Eighteen tumours had sufficient post-treatment frozen material to allow analysis with the yeast functional assay after chemotherapy. Among them, all 12 tumours that did not bear *TP53* mutation before treatment did not show post-treatment *TP53* mutation.

Six out of nine tumours with *TP53* mutation before treatment had good quality post-treatment tissue available and have been further analysed for post-treatment *TP53* status (Table 4). Among these six tumours, two still bore a mutation after treatment (patients 3 and 12), whereas no mutation could be detected by the functional test in four others patients (patients 7, 8, 9 and 11). For these four patients with absence of detectable *TP53*-mutated cells after treatment, note that three of them had either a dense inflammatory stroma (patients 9 and 11) or a single small (2 mm) residual focus of tumour cells (patient 8).

### Allelic profiles and clinicopathological data

The distribution of the 11 post-chemotherapy profile changes was not linked to any other clinicopathological parameter (age, tumour grade, tumour stage, ESR1 status, ERBB2 status and type of incomplete pathological response) and was not linked to the pre-treatment *TP53* status of the tumours.

## DISCUSSION

We found that genetic changes are detectable early after chemotherapy in breast cancers.

All 29 patients had poor prognosis breast tumours treated with the same dose–dense epirubicin/cyclophosphamide regimen. The large amount of tumour tissue obtained before (open incisional biopsy) and after (mastectomy) treatment allowed reliable assessment of response to treatment. Obtaining such homogeneous pre- and post-chemotherapy tissue samples is difficult and explains why we could study only 29 patients.

Advanced breast cancers are often high grade with necrotic areas, and become more heterogeneous after treatment, with inflammatory infiltrate, oedema, fibrosis and necrosis. To overcome tissue heterogeneity, we used laser tissue microdissection, which allows a targeted sampling of tumour cells with very little contamination. In this regard, 20/29 tumours in our study had at least one allelic loss greater than 80%, indicating that tumour tissue samples were made of almost pure tumour cells. Although laser-microdissection is a highly efficient sampling method, it yields limited amounts of tissue material, and we took special care of sampling at least 5000 cells for each tumour. Indeed, allelic profiling must take into account allelic dropout artefacts that are artificial allelic imbalances occurring when DNA concentration is too low (Coulet *et al*, 2000; Miller *et al*, 2002). Several tumour areas were sampled in each tumour to obtain a full representativity of the tumour cell population and to avoid the sampling of genetically different subclones. In addition, all PCR showing important differences between pre- and post-treatment allelic profiles were confirmed with a second round of PCR using microdissected material and a third PCR with non-microdissected tumour tissue.

The chemotherapeutic regimen used in this study contained epirubicin and cyclophosphamide that are both well known for their cytotoxic and mutagenic effects *in vitro*. Anthracyclins are topoisomerase II inhibitors that induce DNA double-strand breaks (Ferguson and Baguley, 1996). In animal or human cell cultures, anthracyclins induce chromosomal alterations rather than mutations (El-Mahdy Sayed Othman, 2000; Baumgartner *et al*, 2004), often through homologous recombination mechanisms (Lehmann *et al*, 2003). The altered cells then either engage their apoptotic programme or accumulate genetic alterations (Morris *et al*, 1995). Alkylating agents transfer alkyl groups to cellular constituents, inducing DNA adducts as well as DNA crosslinking that may lead to point mutations (Sanderson and Shield, 1996; Hunter *et al*, 2006). A recent study using microarray-based CGH on 21 breast tumours before and after epirubicin/cyclophosphamide chemotherapy showed a significant acquired copy number gain on 11p15.2–11p15.5 after treatment (Pierga *et al*, 2007). In 30 patients with breast cancers treated with alkylating agents, microsatellite instability and LOH were found in peripheral blood mononuclear cells, respectively, in 27 and in 6 patients (Fonseca *et al*, 2005), illustrating how acute chemotherapy-induced DNA damage can also affect normal cells.

In our study, at least two different mechanisms could explain the increase of AI at 11p15 and 11q23.3 after chemotherapy: a pre-existent undetectable small population of tumour cells bearing one AI could have been selected by the treatment that would have preferentially targeted the tumour cells without AI. Alternatively, new AI could be directly induced by the treatment in a sufficient number of tumour cells to become detectable through molecular analyses. In this regard, changes induced by anthracyclins at the 11q23 *MLL* locus lead to therapy-related acute leukaemias (Allan and Travis, 2005). We also observed in two tumours, at D13S171 (13q13.1) (patient 6) and D16S496 (16q22.1) (patient 39), imbalance on one allele before chemotherapy followed by imbalance on the other allele after chemotherapy. These ‘inversed profiles’ could be explained by selection of a minor subclone bearing one allelic loss through a preferential effect of the drug on a major subclone bearing loss of the other allele. In seven PCR (six tumours, five markers: D7S490 (7q31.32), D8S261 (8p22), D11S1356 (11q23.3), D13S171 (13q13.1) and IGP53 (17p13.1), AI present before treatment strongly decreased after treatment, leading to almost normal allelic profiles in five cases. Contamination by non-tumour cells may hide AI in tumour cells, but we purposely used laser microdissection to prevent this bias. The most likely explanation for these ‘improvements’ is chemotherapy-driven selection of tumour subclones without AI.

One purpose of our study was to determine whether post-chemotherapy genetic abnormalities could occur at different rates in *TP53*-mutated versus *TP53*-nonmutated breast tumours. *TP53* mutations are observed in 20 to 30% of breast carcinomas and are linked to poor prognosis (Turpin *et al*, 1999; Olivier *et al*, 2006). Loss of TP53 function may result in increased genetic instability with higher frequency of mutations, chromosomal abnormalities, gene amplifications, LOH and abnormal chromosome segregation (Fukasawa *et al*, 1996; Morris, 2002). Our study failed to demonstrate strong link between the pre-treatment *TP53* status of the tumours and the occurrence of post-treatment genetic events. Further investigations using xenografted human breast tumours would allow sequential analyses of the genetic alterations during chemotherapy-induced tumour regression. Yet in four cases (patients 7, 8, 9 and 11), the *TP53* mutation found before chemotherapy was not found after (Table 4). This could indicate, as in our previous observations, that the *TP53*-mutated cells were targeted by the chemotherapy, especially in one case (patient 7) that showed a low post-treatment stromal cell density as well as an important decrease of the proportion of cells with *TP53* LOH (Table 3). The other three post-treatment tumours (patients 9, 11 and 8) had either a high stromal cell density (patients 9 and 11) or consisted of a single small residual nodule (patient 8) that might have impeded detection of the *TP53* mutation in these non-microdissected frozen samples. Immunohistochemistry showed either negative stainings or a trend to a decrease in the proportion of TP53-overexpressing cells after treatment, pointing to higher chemosensitivity of *TP53*-mutated cells, even if TP53 immunohistochemistry is a less reliable indicator of the *TP53* gene status than the yeast functional assay.

In this study, we demonstrate that genetic changes do occur early after chemotherapy in breast carcinomas. The most likely explanation for our observations is chemotherapy-driven selection of tumour subclones: breast cancer tumour cells are well known to be highly heterogeneous (Shipitsin *et al*, 2007) and those already bearing genetic alterations may be more sensitive to chemotherapy and enter apoptosis more rapidly than unaltered cells. Another recent report also showed that such post-treatment changes do exist but, as in our study, seem to be rare (Pierga *et al*, 2007). Whether specific molecular abnormalities can facilitate or impede response to therapy is a crucial issue in oncology (Tanner *et al*, 2006). However, exquisite chemosensitivity linked with specific allelic losses in tumours has not been reported yet. The disappearance of *TP53*-mutated cells after chemotherapy in one case (patient 7) is clearly in favour of clonal selection. Yet, whatever the mechanism, these treatment-induced events should favour the emergence of therapy-resistant tumour subclones and subsequent tumour recurrences.

## Figures and Tables

**Table 1:** Clinicopathological data for the 29 patients

	***n* (%)**	**Absence of response**	**Minor response**	**Major response**
*Age*				
⩽50	22 (76)	3	14	5
>50	7 (24)	1	3	3
				
*Tumour stage*				
IIa	1 (3)	—	—	1
Ilb	3 (10)	1	1	1
IIIa	13 (45)	1	9	3
IIIb	8 (28)	1	6	1
IV	4 (14)	1	1	2
				
*Histological tumour type*				
Ductal	25 (86)	2	16	7
Lobular	3 (10)	1	1	1
Mucinous	1 (4)	1	—	—
				
*Tumour grade*				
2	14 (48)	2	11	1
3	15 (52)	2	6	7
				
*ESR1*				
Neg	8 (28)	2	1	5
Pos	21 (72)	2	16	3
				
*ERBB2*				
Neg	23 (79)	3	16	4
Pos	6 (21)	1	1	4
				
*TP53 status*				
Not mutated	20 (69)	3	14	3
Mutated	9 (31)	1	3	5
*Total*	29	4	17	8

*n*, number of patients; ESR1, oestrogen receptor alpha.

**Table 2:** Characteristics of the ten microsatellites analysed: results of the 29 patients are compared to the literature data

**Locus**	**Chromosomal region**	**Length of PCR product**	**% of LOH in the chromosomal region in breast tumours in the literature**	**LOH at the defined locus in informative cases before chemotherapy in this study**	**LOH at the defined locus in informative cases after chemotherapy in this study**
D3S1573	3p21.31	136–154	21–41% (Braga *et al*, 1999)	6/18 (33%)	7/19 (37%)
D7S490	7q31.32	92–106	11% (Mao *et al*, 2005)	5/26 (19%)	5/25 (20%)
D8S1820	8p21.1	103–117	29% (Wang *et al*, 2004)	10/20 (50%)	9/19 (47%)
D8S261	8p22	128–144	52% (Hirano *et al*, 2001); 26% (Wang *et al*, 2004)	13/23 (57%)	10/24 (42%)
D11S860	11p15	154–196	28% (Hirano *et al*, 2001)	5/14 (36%)	5/11 (45%)
D11S1356	11q23.3	193–213	47% (Hirano *et al*, 2001); 50% (Wang *et al*, 2004)	14/23 (61%)	11/21(52%)
D13S171	13q13	227–231	34% (Miyakis and Spandidos, 2002)	9/18 (50%)	6/18 (33%)
D16S496	16q22.1	209–226	42% (Shen *et al*, 2000)	6/23 (26%)	10/22 (45%)
IGP53	17p13.1	97–138	51% (Hirano *et al*, 2001); 56% (Wang *et al*, 2004)	9/23 (39%)	6/20 (30%)
D17S855	17q21.31	143–155	34% (Hirano *et al*, 2001); 43% (Mao *et al*, 2005)	12/24 (50%)	10/24 (42%)

**Table 3:**
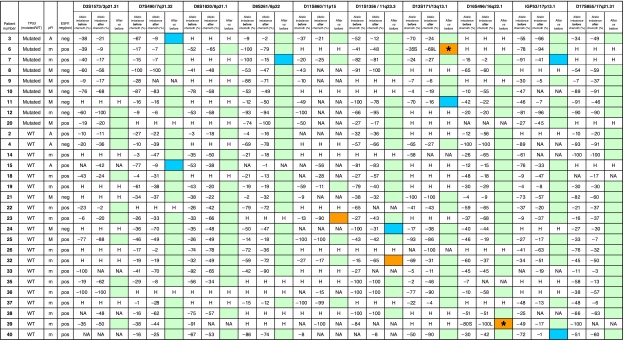
Allelic profiles before and after chemotherapy in 29 tumours with or without *TP53* mutation

**Table 4:** *TP53* status before and after treatment in the nine *TP53*-mutated tumours

**Patient number**	**TP53 gene status^a^ before treatment**	**TP53 gene status^a^ after treatment**	**TP53 IHC before treatment**	**TP53 IHC after treatment**
3	Mut 82% (Codon 175, R *vs* H)	Mut 93% (Codon 175, R *vs* H)	30%	10%
12	Mut 23% (Codon 158, Insert - frameshift)	Mut 30% (Codon 158, Insert - frameshift)	Negatif	Negatif
7	Mut 22% (Codon 144, Q *vs* STOP codon)	WT	Negatif	Negatif
8	Mut 96% (Codon 220, Y *vs* C)	WT	50%	10%
9	Mut 54% (Codon 273, R *vs* H)	WT	20%	20%
11	Mut 20% Codon 317, Q *vs* STOP codon)	WT	Negatif	Negatif
6	Mut 63% (Codon 248, R *vs* W)	NA	80%	50%
10	Mut 30% (Codons 131 to 133, deletion- frameshift)	NA	Negatif	Negatif
20	Mut 62% (Codon 193, H *vs* R)	NA	40%	Negatif

IHC, immunohistochemistry; Mut, mutated; NA, not available; WT, wild type.

a Per cent of mutated yeast colonies (mutation type).
